# Methyl Donor Micronutrients: A Potential Dietary Epigenetic Target in Systemic Lupus Erythematosus Patients

**DOI:** 10.3390/ijms24043171

**Published:** 2023-02-06

**Authors:** Karen Pesqueda-Cendejas, Bertha Campos-López, Paulina E. Mora-García, José M. Moreno-Ortiz, Ulises De la Cruz-Mosso

**Affiliations:** 1 Red de Inmunonutrición y Genómica Nutricional en las Enfermedades Autoinmunes, Centro Universitario de Ciencias de la Salud, Universidad de Guadalajara, Guadalajara 44340, Jalisco, Mexico; 2 Instituto de Neurociencias Traslacionales, Departamento de Neurociencias, Centro Universitario de Ciencias de la Salud, Universidad de Guadalajara, Guadalajara 44340, Jalisco, Mexico; 3 Programa de Doctorado en Ciencias de la Nutrición Traslacional, Centro Universitario de Ciencias de la Salud, Universidad de Guadalajara, Guadalajara 44340, Jalisco, Mexico; 4 Programa de Doctorado en Ciencias en Biología Molecular en Medicina, Centro Universitario de Ciencias de la Salud, Universidad de Guadalajara, Guadalajara 44340, Jalisco, Mexico; 5 Instituto de Genética Humana “Dr. Enrique Corona Rivera”, Departamento de Biología Molecular y Genómica, Centro Universitario de Ciencias de la Salud, Universidad de Guadalajara, Guadalajara 44340, Jalisco, Mexico

**Keywords:** systemic lupus erythematosus, methyl donor nutrients, DNA methylation, miRNAs, histone modifications, epigenetic diet

## Abstract

Systemic lupus erythematosus (SLE) is a chronic autoimmune disease characterized by an aberrant immune response and persistent inflammation. Its pathogenesis remains unknown; however, a complex interaction between environmental, genetic, and epigenetic factors has been suggested to cause disease onset. Several studies have demonstrated that epigenetic alterations, such as DNA hypomethylation, miRNA overexpression, and altered histone acetylation, may contribute to SLE onset and the disease’s clinical manifestations. Epigenetic changes, especially methylation patterns, are modifiable and susceptible to environmental factors such as diet. It is well known that methyl donor nutrients, such as folate, methionine, choline, and some B vitamins, play a relevant role in DNA methylation by participating as methyl donors or coenzymes in one-carbon metabolism. Based on this knowledge, this critical literature review aimed to integrate the evidence in animal models and humans regarding the role of nutrients in epigenetic homeostasis and their impact on immune system regulation to suggest a potential epigenetic diet that could serve as adjuvant therapy in SLE.

## 1. Introduction

Systemic lupus erythematosus (SLE) is a chronic autoimmune disease characterized by a loss of tolerance to self-antigens and, consequently, overactivation of B and T cells, which promote an aberrant immune response and chronic inflammation. Innate and adaptative responses against self-antigens induce autoantibodies production, a key mechanism related to SLE manifestations, particularly when they form immune complexes. The deposition of the immune complex activates the immune system by complement activation and by Fc receptor binding, which can damage different organs: skin, kidneys, joints, lungs, coronary system, and liver [1,2]. The incidence of SLE varied from 0.3–23.7 per 100,000 person-years, with a prevalence of 20–70 per 100,000 depending on racial background, socioeconomic factors, and environmental exposure [3,4].

The sequence of events that lead to autoimmunity remains unknown; however, the evidence suggests a complex interaction between genetic and environmental factors, and epigenetic modifications have also been suggested as a risk factor for SLE susceptibility [1,5].

The first person to establish the field of epigenetics was Conrad Hal Waddington, who coined the term and again observed the non-Mendelian inheritance phenomenon with *Drosophila melanogaster* [6]. The term epigenetic refers to hereditable changes in gene function that occur without changes in DNA sequence, including post-translational modifications, such as phosphorylation, ubiquitination, biotinylation, sumoylation, ADP ribosylation, acetylation, and methylation of histones [7]. Regarding SLE, non-coding RNAs (ncRNAs) and changes in chromatin structure due to altered DNA methylation are epigenetic mechanisms involved in its pathogenesis [1,8]. In mammals, DNA methylation is primarily a stable mark found at cytokines in CpG dinucleotides; however, its regulation is a dynamic process that may occur in a gene-specific and global manner [9]. Approximately 70% of vertebrate gene promotors are associated with DNA elements called CpG islands, which integrate the activity of a range of chromatin-regulating factors that post-translationally modify and modulate gene expression [10].

Diet is a relevant player in epigenetic homeostasis. The epigenetic mark most widely described is methylation, probably due to levels of S-adenosyl-methionine (SAM), a methyl donor that is dependent on dietary micronutrients, such as folate, methionine, choline, and B vitamins, such as B6 and B12 [1]. Evidence demonstrates that altered consumption of these nutrients acts to modify global methylation and in the promoters of disease-related genes in animals and humans [9].

In SLE, global and specific hypomethylation in CD4^+^ T lymphocytes has been associated with clinical disease activity, while hypomethylation of B cells is related to the SLE outset [5]. Epigenetic changes could be influenced by environmental factors, such as diet, which provides a feasible intervention for autoimmune diseases. Based on this knowledge, this literature review aimed to integrate the evidence in animal models and humans regarding the role of nutrients in epigenetic homeostasis and their impact on immune system regulation to suggest a potential epigenetic diet that could serve as adjuvant therapy in SLE.

## 2. Epigenetic Mechanisms Related to SLE Pathophysiology

Epigenetic events are regulatory mechanisms that control the accessibility of chromatin to transcriptional regulatory elements and can be influenced by several environmental factors that are dynamic and heritable [11]. Regarding SLE, it has been suggested that epigenetic mechanisms contribute to the dysregulation of innate and adaptive immune responses; the pathophysiology of SLE is not fully understood, but the evidence indicates the participation of epigenetic alterations in effector lymphocyte generation dysregulated cytokine expression and tissue damage [11].

### 2.1. DNA Methylation and SLE

DNA methylation is an epigenetic process with cytosine methylation in CpG sites, which results in gene silencing in the regulatory region. The methylated status of CpG sites in a promoter region generally blocks accessibility to transcriptional factors that inhibit gene transcription. The methylation status of DNA depends on three kinds of enzymes: DNA methyl transferase 1 (DNMT1) exerts maintenance methylation, methyl CpG-binding domain 2 (MBD2) is related to the demethylation effect, and DNA methyl transferase 3 (DNMT 3A and DNMT 3B) is related to the novo methylation [12].

Changes in DNA methylation can affect the expression of genes involved in the immune response; therefore, global methylation may play a relevant role in autoimmune diseases, such as SLE [12]. A reduced expression and activity of DNMTs are related to hypomethylation and active demethylation mediated by *ten-eleven translocation* proteins (TETs), which could influence changes in DNA methylation and alter the expression of genes involved in the immune response; in this sense, it was previously reported that hypomethylation of IFN-related genes such as *IFI44L* and *BST2* correlated with overexpression of these genes in total CD4^+^ T cells from lupus patients [13]. Similarly, Integrin Subunit Alpha L (*ITGAL*) (CD11a) promoter hypomethylation was found in T cells from SLE-active patients [14], indicating a possible role in SLE pathogenesis.

**Figure 1:**
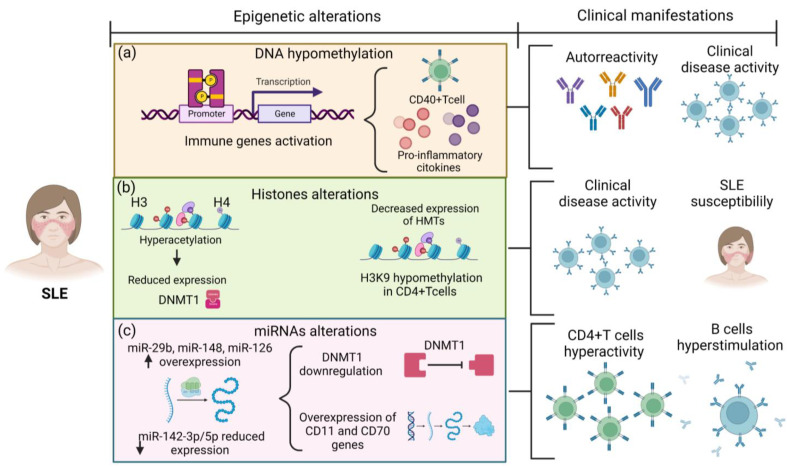
Epigenetic alterations in the pathophysiology of SLE. (**a**) SLE patients present epigenetic alterations as DNA hypomethylation of immune-related genes promoting immune cell overexpression and pro-inflammatory cytokine overproduction, which is associated with autoreactivity and clinical disease activity on SLE. 1. Red circles: Interleukins-13; Purple circles: Interleukins-10. (**b**) H3 and H4 histone hyperacetylation reduces DNMT1 expression; additionally, H3K9 hypomethylation in CD4+ T cells is associated with clinical disease activity and SLE susceptibility. (**c**) The overexpression of miR-29, miR-148, and miR-126 induces DNMT1 downregulation; in contrast, reduced expression of miR-142-3p/5p promote overexpression of CD11 and CD70 genes. These miRNA disturbances result in CD4+ T cell hyperactivity and B cell hyperstimulation. SLE: systemic lupus erythematosus patients; DNMT1: DNA methyltransferase.

Balada et al. reported that in T cells, DNA hypomethylation might alter the expression of genes that could induce autoreactivity [15]. In SLE, the evidence confirmed that those patients presented hypomethylation in CD40^+^ T cells which contributes to the overexpression of SLE-related genes such as CD11a, CD70, and CD154/CD40 ligand (CD40L) [16]. In addition, hypomethylation of the *CD40L* promoter was associated with the clinical disease in female SLE patients [17] (Table 1 and Figure 1). On the other hand, levels and expression of cytokines, such as interleukin 10 (IL-10) and interleukin 13 (IL-13), were correlated with SLE clinical disease activity. According to Zhao et al., the methylation status of the regulatory regions of *IL-10* and *IL-13* genes was reduced in SLE patients compared to controls (Figure 1). IL-13 is involved in T helper 2 cell (Th2) differentiation and stimulates the proliferation and differentiation of B lymphocytes by inducing IgM and IgG production. These results highlight the critical role of DNA methylation in regulating the expression of Th2 cytokines in SLE, which may also influence the clinical disease activity in these patients [18] (Table 1).

### 2.2. ncRNAs and SLE

Studies support that methylation inhibition is able by itself to induce the onset and progression of SLE. The exact mechanisms that promote hypomethylation in SLE are not fully understood; however, microRNAs (miRNAs) have been suggested as mechanisms capable of inhibiting the DNA methylation machinery [19,24]. miRNAs are small non-coding, single-stranded RNA molecules with a length of 19 to 25 nucleotides that regulate gene expression at the posttranscriptional level by degrading or blocking the translation of messenger RNA (mRNA) [25].

In SLE, miRNA dysregulation has been described, affecting innate and adaptative responses, exacerbating T and B cell activity, and producing excessive inflammatory cytokines [24]. Increased expression of miR-126-3p, miR-7, and miR-326 and decreased expression of miR-31 and miR-146a, a negative regulator of type 1 IFN signaling, was observed in SLE peripheral blood mononuclear cells (PBMC). In purified B cells from SLE patients, miR-150, miR-16, miR-15, and miR-155 were overexpressed compared to healthy controls [13], suggesting that mechanisms involving specific miRNAs could influence SLE pathogenesis.

Moreover, the link between miRNAs and DNA methylation was described by Qin et al. They demonstrated that overexpression of miR-29b induced downregulation of DNMT1, which was related to hypomethylation in SLE (Figure 1) [19]. Similarly, the enhanced expression of miR-21, miR-148, miR-126, and miR-29b in CD4^+^ T cells negatively regulates DNMT1, which is associated with hypomethylation and overexpression of sensitive-methylation genes, such as CD11a and CD70 [19,21] (Table 1). In contrast, the reduced expression of miR-142-3p/5p in CD4+ T cells was associated with CD4+ T cell hyperactivity and B cell hyperstimulation in SLE patients [21] (Table 1) (Figure 1).

### 2.3. Histones Post-Translational Modifications and SLE

Histones are proteins that wrap DNA to form nucleosome structures. Residues located at histone tails, most commonly lysine and serine residues, are susceptible to post-translational and covalent modification, such as phosphorylation, ubiquitination, acetylation, and methylation; these modifications are catalyzed by histone-modifying enzymes.

Histone acetyltransferases (HATs) catalyze the addition of an acetyl group to the histone’s tails, contributing to the accessibility of transcriptional factors to chromatin; therefore, histone acetylation is associated with gene expression activation; in contrast, histone deacetylases (HDACs) remove acetyl groups to inhibit it [26]. Histone methyltransferases are other histone-modifying enzymes that modify proteins by adding a methyl group, which may decrease chromatin accessibility and reduce gene expression [26].

Specifically, histone 3 lysine 8 acetylation (H3K18ac) is related to conferring chromatin “opening”, while histone H3 lysine 9 methylation (H3K19me3) regulates chromatin condensation and transcriptional silencing [27].

Regarding histone acetylation, acetyl-CoA can act as a metabolic signal by promoting the activity of specific HATs, whereas NAD^+^ levels regulate the activity of sirtuin 2, a HDAC dependent on NAD^+^ [28].

It has been shown that histone status could regulate inflammatory and anti-inflammatory genes by determining their activation state [26]; therefore, histone modifications could play a relevant role in autoimmunity onset.

An altered state of histone modifications has been suggested in SLE. Gautam et al. showed that SLE patients presented H3 and H4 hyperacetylation with a decrease in DNMT1 expression compared to healthy controls; elevated histone H3 acetylation has also been correlated with the clinical disease activity in SLE [22,29] (Table 1) (Figure 1). In addition, Fang et al. described that SLE patients had higher methylation and thus lower expression in the histone deacetylases 6 (*HDAC6*) promoter than healthy controls; decreased HDAC6 levels could result in increased histone acetylation and high immune-related gene expression; therefore, the authors suggested that may be related to SLE susceptibility [23] (Table 1).

Regarding histone methylation, this mechanism is catalyzed by HMTs by adding methyl groups to arginine and lysine histone residues, while HDMTs remove them [26]. Altered histone methylation has been described in SLE; apparently, these patients presented decreased expression of some HMTs with global histone H3K9 hypomethylation in SLE CD4^+^ T cells in humans and murine models (Figure 1). Nevertheless, the mechanism related to altered histone methylation in SLE remains unknown [30].

## 3. Nutrients’ Role in Epigenetic Modifications

Epigenetic changes are dynamic and susceptible to modification in response to environmental stimuli, which provides a feasible intervention to influence epigenetic homeostasis through an “epigenetic diet.” The term epigenetic diet was previously described by Daniel et al., referring to the consumption of certain foods, such as soy, grapes, vegetables, and green tea, which exert mechanisms against aging and cancer. They also suggested that introducing these food groups into a regular diet regime could serve as an effective therapeutic strategy for medical and chemo-preventive purposes [7]. In this sense, a diet source of micronutrients such as folate, choline, B vitamins, and methionine that acts as methyl donors or directly influences DNA methylation, such as fatty acids, may provide the possibility of designing an epigenetic diet in SLE treatment (Table 2).

### 3.1. Folate

Folate, also known as vitamin B9, is an essential micronutrient vital for cellular functions, such as DNA synthesis and methylation [37]. Its immune functions have been previously described. According to Courtermanche et al. [38], dietary folate deficiency is likely to affect most dividing cells and could cause DNA breaks in human T lymphocytes, affecting their proliferation and increasing apoptosis rate. Similarly, folate deficiency affects cell function and T helper cell differentiation, which suggests that folate plays a relevant role in maintaining Th cell homeostasis [39]. In this sense, folate may directly influence the immune system by influencing immune cell homeostasis and indirectly by influencing the DNA methylation of immune genes.

**Figure 2:**
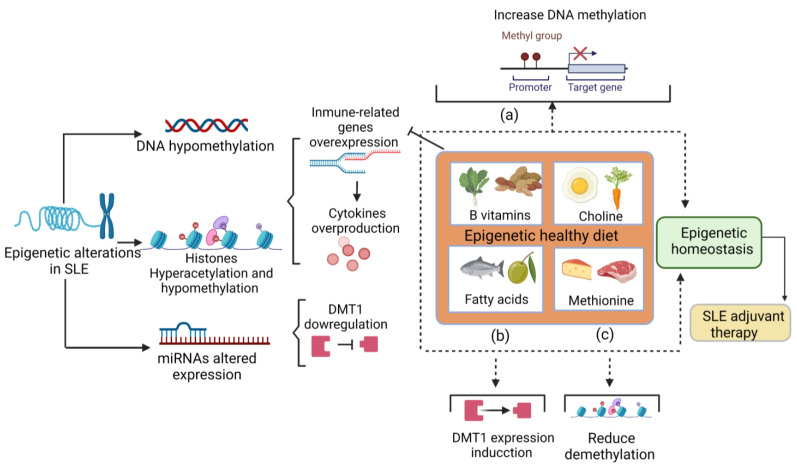
The role of the diet in epigenetic changes. Nutrients have a role in epigenetic modifications. (**a**) B vitamins and choline donate methyl groups to promote DNA methylation and could reduce the expression of immune-related genes and subsequently reduce cytokine overproduction. (**b**) Fatty acids stimulate DNMT1 expression, which could compensate for the downregulation of DNMT1 in SLE patients. (**c**) The methionine intake reduces hydroxymethylation a step before demethylation. Altogether, these mechanisms promote epigenetic homeostasis. Therefore, a healthy epigenetic diet that provides an adequate amount of these nutrients acts as an adjuvant therapy for SLE. SLE: systemic lupus erythematosus. DNMT1: DNA methyltransferase 1.

Under normal dietary conditions, the folate absorbed is metabolized to its bioactive form of 5-methyltetrahydrofolate (5-methylTHF); this metabolite is required to maintain the flux of methyl groups for the re-methylation of homocysteine to methionine. Methionine is the substrate for SAM or S-adenosyl-L-methionine (AdoMet), a methyl group donor for methylation reactions that most commonly occurs at the 5 positions of cytosine to generate 5-methylcytosine [40]. The evidence suggests that folate-deficient diets may induce DNA hypomethylation. A study conducted on women aged 65–80 reported that inadequate folate intake during 7 weeks resulted in DNA hypomethylation in leukocytes [31], which highlights the role of folate in the modulation of DNA methylation (Figure 2).

In humans and rats, hypomethylation may be induced by low dietary folate and reversed by folate repletion. In a randomized controlled trial, supplementation with 400 µg per day of folate led to an increase in DNA methylation of 31% in leucocytes and 25% in colonic mucosa; however, the statistical effect was marginal [31]. In a similar study, DNA hypomethylation by folate repletion was reversed with supplementation of 286–516 µg for 3 weeks (Table 2) [32]. Notably, DNA hypomethylation reversibility depends on folate depletion duration. In a study conducted in rats with folate deficiency for 9, 18, 24, and 36 weeks, the repletion of the adequate folate diet just increased the DNA methylation in those with 9 weeks of folate depletion [41], which suggests that hypomethylation is not reversibly after prolonged folate deficiency.

### 3.2. Choline and Betaine

Choline is an essential nutrient involved in synthesizing the neurotransmitter acetylcholine, methyl group donor, betaine, and phospholipids. It has also been associated with preventing cognition alterations, hepatic steatosis, cardiovascular disease, and cancer. Choline also has a relevant role in epigenetic regulation through SAM synthesis (Figure 2) during its oxidation to betaine [42].

Studies of global and gene-specific DNA methylation in rodent models exposed to a maternal choline-deficient diet showed global and specific hypomethylation of cyclin-dependent kinase 3 (CDKN3), calbindin1, and vascular endothelial growth factor (VEGF-C) genes. Notably, this study also observed global hypomethylation but gene-specific hypermethylation of insulin-like growth factor-2 (IGF-2) [43]. This demonstrates that methyl donor nutrients could act differently by depending on global or gene-specific methylation and by depending on the gene evaluated; however, more studies are necessary to prove this theory. Another similar study has demonstrated that Maternal choline supply modifies fetal histone and DNA methylation in rat fetal liver and brain (Table 2), suggesting that choline exerts epigenetic mechanisms during stages of embryonic development [33].

Betaine is a nonessential nutrient found in several food sources and can be synthesized from choline; it has an essential role in DNA methylation, playing as a methyl donor. Recently, it has been suggested that betaine plays an anti-inflammatory role through the betaine NF-κB signaling pathway, and NLRP3 inflammasome inhibition [44]. Due to its epigenetic and anti-inflammatory effects, adequate choline and betaine intake should be ensured in SLE patients.

### 3.3. B Vitamins

B vitamins comprise a group of water-soluble vitamins that perform essential cellular functions in catabolic and anabolic enzymatic reactions [45] but also play a relevant role in epigenetic regulation, acting as methyl donors or as cofactors on the one-carbon metabolism (Figure 2) [46].

Vitamin B12, also known as cobalamin, in one-carbon metabolism acts as a cofactor for the enzyme methionine synthase; thus, it is a regulator of DNA methylation [46]. Cobalamin deprivation induced global hypomethylation in a murine model, even when combined with a folate-rich diet (Table 2) [34]. This indicates that the high consumption of other methyl donors cannot compensate for the cobalamin deficiency. Vitamin B12 could also directly affect the immune system; its deficiency has been associated with TNF-alfa overproduction [47]; thus, adequate cobalamin consumption may have anti-inflammatory effects on SLE.

Pyridoxine or vitamin B6 serves as a coenzyme in the transfer of a one-carbon unit from serine to tetrahydrofolate (THF) to generate glycine and 5,10 methylenetetrahydrofolate (MTHF) [46]. Moreover, its role in methylation, it has been reported that B6 deficiency results in inflammation and has been involved in inflammatory diseases, such as rheumatoid arthritis [48]; however, there is no evidence of its specific role in SLE.

Riboflavin has potent anti-inflammatory and antioxidant effects; its deficiency has been associated with disturbing MTHFR activity and to the risk of cancer by influencing DNA methylation patterns. In SLE, pyridoxine has been inversely associated with atherosclerotic plaque, a common alteration in these patients; there is no evidence of pyridoxine and DNA methylation in SLE; however, an adequate intake of this vitamin may reduce cardiovascular alterations in SLE and avoid disturbances of the MTHFR activity [49,50].

### 3.4. Methionine

Methionine is an essential sulfur-containing amino acid that must be consumed in the diet. Eggs, fish, dairy products, and some meats are sources of it. Regarding its role in DNA methylation, it serves as a precursor of SAM; thus, when the concentration of methionine is low, SAM synthesis is reduced, and DNA methylation is theoretically reduced [43]. A study conducted on 168 women of reproductive age showed that high methionine intake reduces hydroxymethylation (a step that precedes demethylation) (Table 2) (Figure 2) [35]. However, methionine’s biological effects are controversial because it is necessary to preserve DNA methylation patterns; however, in cancer, evidence suggests that methionine restriction inhibits cancer cell growth and may enhance the efficacy of chemotherapeutic agents. In addition, methionine has been involved in oxidative and aging events [51,52]. Due to these possible adverse effects of methionine, it needs to be clarified how to be the recommendation for methionine intake in SLE patients; however, an adequate but not excessive amount of methionine may be included in the dietary recommendations for SLE.

### 3.5. Fatty Acids

Fatty acids are no part of the methyl donor nutrients; however, emerging findings support that fatty acids can modify the epigenome. Until now, the exact mechanisms through which fatty acids influence epigenetic changes were not known; however, some mechanisms have suggested that short-chain fatty acids, such as butyric, propionic, and valeric, can inhibit histone deacetylase activity. On the other hand, variation in energy intake led to changes in cellular NAD+/NADH which may alter histone deacetylase activities; therefore, indirectly, fatty acid could modulate histone deacetylase activity through energy changes [53].

The evidence in the murine model and humans reported that PUFAs and saturated fatty acid intake might alter the methylation status; however, the specificity of such effects still needs to be clarified [53]. In human colorectal cancer cells, PUFA treatment increases the expression of DNMTs in HT29/219 (Figure 2) but suppresses other cell lines (Table 2) [36]. Thus, more evidence about fatty acids and their epigenetic roles is necessary.

Regarding the immune system, PUFAs, specifically omega-3 fatty acids, have been widely related to immune function due to their anti-inflammatory properties. In macrophages, treatment with omega-3 decreases pro-inflammatory cytokine synthesis and increases IL-10 cytokine production [54]. Additionally, omega-3 fatty acid promotes M2 polarization in murine models and may modulate T cell activation. Even omega-3 fatty acid supplementation has demonstrated beneficial effects in T-mediated diseases, such as autoimmune hepatitis [54] and probably SLE.

### 3.6. Other Environmental Factors That Impact DNA Methylation

Chronic alcohol consumption could promote DNA hypomethylation; it has been demonstrated that alcohol can inhibit methionine synthase activity in the liver, resulting in a significant reduction in s-adenosyl methionine levels [55].

In addition, tobacco compounds can promote cobalamin and folate inactivation and interfere with one-carbon metabolism, consequently interfering with the availability of methyl groups [43]. Therefore, promoting healthy habits in SLE patients, such as avoiding or at least limiting alcohol intake and smoking, is crucial in treating the disease.

## 4. Diet as an Epigenetic Therapy in SLE: A New Paradigm?

The impact of dietary interventions may determine the curse of SLE pathogenesis; for instance, increased methylation status through a rich methyl donor diet is associated with reducing clinical disease activity in SLE patients [56]; in contrast, the restriction of methionine, one of the principal methyl donor nutrients, is related to reducing aging effects through oxidative stress reduction in healthy individuals [52]. This brings to light a new “paradigm” about how diet in SLE patients must be; the research on SLE has been increasing in recent decades, but there is still no consensus about specific dietary recommendations that allow attenuating SLE clinical manifestations. However, the intake of nutrients involved in epigenetic mechanisms based on the Dietary Reference Intake (DRI) should be part of SLE nutritional recommendations (Table 3).

### A Diet to Increase Methylation in SLE

The association between dietary product consumption and DNA methylation was described by a study conducted on 61 female SLE patients from Germany, where they showed that the consumption of some methyl donor micronutrients such as methionine and choline were associated with CD40L methylation in T cells, and thereby was negatively associated with clinical disease activity. Nevertheless, the dietary products with the highest impact on methylation included meat, ice cream, white bread, and cooked potatoes, which could be considered unhealthy [56]. Hence, an epigenetic diet that promotes methylation should be applied carefully and balanced against side effects on other comorbidities, such as obesity and cardiovascular disease.

Based on this, it is necessary to implement not just an epigenetic diet but rather a “healthy epigenetic diet” in SLE. A healthy epigenetic diet could be more challenging but possible. According to Quin et al., overexpression of miR-29 in SLE patients from China was associated with altered DNMT1 activity, promoting aberrant DNA methylation [19]. In contrast, in a previous study, the treatment with 100 μM of polyunsaturated fatty acids (PUFAs), such as docosahexaenoic acid (DHA), eicosapentaenoic acid (EPA), and linoleic acid (LA), for 6 days induced the expression of DNMT1 and DNMT3 influencing DNA methylation; though these results were shown in human colorectal cancer cells [36] is well known that a healthy diet includes the adequate consumption of PUFAs; also, there is evidence that suggests a beneficial effect of PUFAs in SLE by reducing inflammatory process [57,58], thus promote the intake of foods source of PUFAs such as vegetal oils like soybean, and sunflower, walnuts, seeds, and marine sources as salmon and tuna may be used as a safe dietary recommendation in SLE [57].

Regarding methyl donor nutrients, folate has demonstrated reverse hypomethylation [41]. Nevertheless, in addition to its epigenetic effect, adequate folate intake may decrease cardiovascular alterations through decreased homocysteine concentrations, by its antioxidant effects, by influencing nitric oxide synthesis, and by increasing antioxidant enzyme activities [59,60]. SLE patients have a high prevalence of cardiovascular disease, which is the main cause of death among those patients [61]. It has also been described that SLE patients have low folic acid levels compared to healthy individuals [62], which could be attributed to a folate-deficient diet [63]; therefore, promoting the high intake of foods source of folates such as green vegetables, sweet peppers, legumes, egg, and red bread should be part of the dietary recommendations in SLE, influencing the intake of methyl donor dietary products and, to prevent or reverse cardiovascular alterations.

The role of methionine is more controversial, although it is part of the main methyl donor nutrients and apparently is also related to aging effects by increasing oxidative stress [52]; however, a diet with an adequate but not excessive amount of methionine could be safe if it is accompanied by a diet rich in folate, which due to its antioxidant effects may compensate for it.

**Figure 3:**
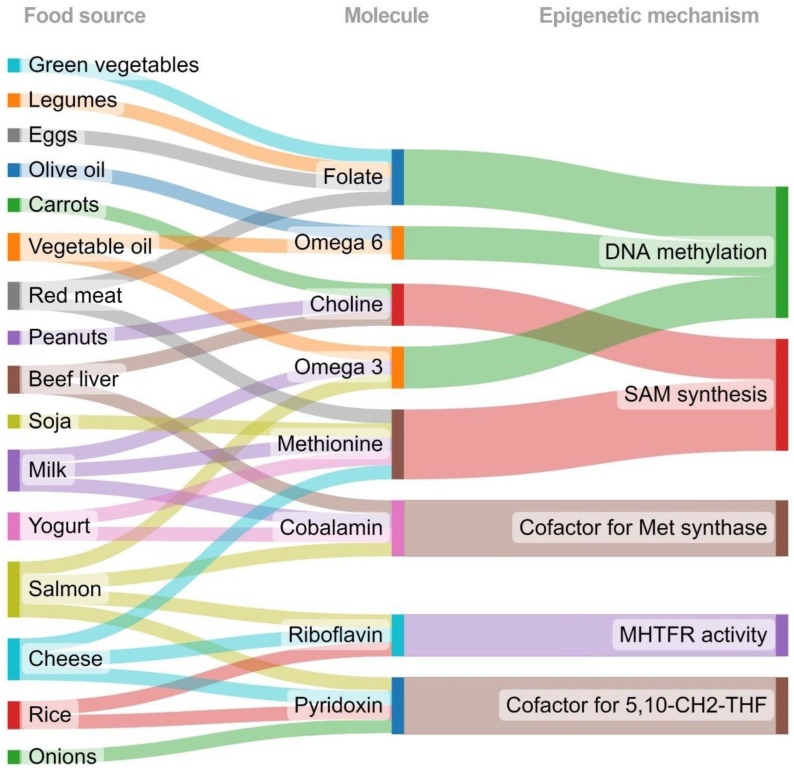
Food sources of molecules involved in epigenetic mechanisms. This diagram graphically illustrates the relationships between the different studies and their characteristics. The nodes’ food sources, molecules, and epigenetic mechanisms show the study characteristics that were compared between the studies. The rainbow lines between the bars reflect the congruencies and differences between the different studies. The wider the gray connection lines, the more congruency that exists. MTHFR: methylenetetrahydrofolate reductase; SAM: S-adenosyl methionine; 5,10-CH2-THF: 5,10 methylenetetrahydrofolate.

B vitamins riboflavin, pyridoxine, and cobalamin are found in various foods, such as meat, whole grains, eggs, dairy products, legumes, nuts, dark leafy vegetables, citric fruits, avocados, and bananas. B vitamins are essential for several biological functions, such as metabolism regulation, hemoglobin synthesis, and maintaining nervous system integrity, but they are also crucial for one-carbon metabolism [43]; accordingly, the intake of these vitamins may induce DNA methylation and gene expression changes that modify the risk of diseases that involve DNA methylation, such as cancer [64], and probably SLE. Nevertheless, due to the lack of clinical trials on methyl donor dietary supplementation in SLE, it may not still be safe to promote it; however, a “healthy epigenetic diet” that provides an adequate amount of methyl donors prevents its deficiency through the intake of common and accessible foods may serve as a viable strategy in SLE treatment. On the other hand, green vegetables, legumes, eggs, and red meat are folate sources and will contribute to DNA methylation. In addition, olive oil, oil vegetables, milk, and salmon provide omega 3 and omega 6, contributing to DNMT expression and promoting DNA methylation. Peanuts and beef liver provide choline, while milk, soja, yogurt, and cheese are methionine sources. Both choline and methionine promote SAM synthesis for one-carbon metabolism. Milk, salmon, and yogurt are foods rich in cobalamin, a cofactor for methionine synthase. Cheese, onions, and rice provide pyridoxine, the cofactor for synthesizing 5,10 methylenetetrahydrofolate. Salmon, cheese, and rice provide riboflavin, which could contribute to MTHFR activity. The Sankey diagram graphically displays these relationships and other characteristics (Figure 3).

## 5. Conclusions

To date, it needs to be clarified how methyl donors will act in a global or in a specific gene way, and it is not possible to target a gene for epigenetic regulation through methyl donor consumption. However, a healthy epigenetic diet rich in food sources of folate, PUFAs, and B vitamins such as riboflavin, pyridoxine, and cobalamin may be a safe coadjutant therapy in SLE patients, with an emphasis on the consumption of common and healthy foods that in turn provide enough methyl donors. As prospects, more in vitro studies are necessary to determine whether nutrients could target specific immune-related genes to modulate their expression through epigenetic mechanisms, such as methylation. Additionally, more evidence based on clinical trials to evaluate methyl donors’ consumption is necessary to establish an epigenetic diet in SLE patients.

## Figures and Tables

**Table 1:** Epigenetic mechanisms related to SLE pathogenesis.

Epigenetic Mechanisms	Change in SLE	Outcomes	References
DNA methylation	Hypomethylation of *CD40L* promotor in CD40+ T cells	Association with clinical disease activity	Vordenbäumen et al., 2021 [17]
	Hypomethylation of *IL-10* and *IL-13* gene regulatory domains	Overexpression of IL-10 and IL-13	Zhao et al., 2010 [18]
ncRNAs	Overexpression of miR-29b	Reduction of DNMT1 levels, DNA hypomethylation, and upregulation of genes encoding CD11 and CD70	Qin et al., 2013 [19]
	Overexpression of miR-126 in CD4+ T cells	Contribution to T cell autoreactivity by targeting DNMT1	Zhao et al., 2011 [20]
	Reduced expression of miR-142-3p/5p in CD4+ T cells	T cell hyperactivity and B cell hyperstimulation	Ding et al., 2020 [21]
Histone modifications	Elevated histone H3 acetylation in CD4+ T cells	Correlation with clinical disease activity	Zhou et al., 2011 [22]
	High methylation in the *HDAC6* promoter	Lower HDAC6 levels and SLE susceptibility	Fang et al., 2016 [23]

SLE: systemic lupus erythematosus; DNMT1: DNA methyltransferase 1; HDAC6: histone deacetylases 6.

**Table 2:** Impact of dietary compounds on DNA methylation.

Dietary Compound	Population/Tissue	Study Design	Outcomes	References
Folate	Colorectal adenoma patients	Randomized controlled trial 400 μg/day for 10 weeks	Increase DNA methylation of 31% in leukocytes and 25% in colonic mucosa	Lu et al., 2006 [31]
	Postmenopausal women	Randomized controlled trial	DNA hypomethylation was reversed with high folate intake (286–516 μg/day)	Jacob et al.,1998 [32]
Choline/betaine	Rat fetal liver and brain	Experimental study Rats fed with several choline doses	Maternal choline supply modifies fetal histone and DNA methylation.	Davison et al., 2009 [33]
Cobalamin (B12)	Wistar rats	Experimental study Rats with and without the absence of B12	Cobalamin deprivation-induced global hypomethylation	Kulkami et al., 2011 [34]
Methionine	Women of reproductive age	Observational cohort study	High intake of methionine in pre-pregnancy reduced hydroxymethylation	Pauwels et al., 2016 [35]
Fatty acids	Human colorectal cancer cells	In vitro study Cells treated with 100 μM of DHA, EPA, and LA for 6 days	Increase the expression of DNMTs in human colorectal cancer cell line HT29/219	Sarabi et al., 2018 [36]

DHA: decosahexaenoic acid; EPA: eicosapentaenoic acid; LA: linoleic acid; DNMTs: DNA methyltransferases.

**Table 3:** Dietary compounds, food source, and dietary recommended intake.

Dietary Compound	DRI	Food Source
Folate	400 µg DFE/day	Green vegetables, sweet peppers, legumes, oranges, eggs, red meat
Choline	400 mg/day	Beef liver, egg, soybeans, potatoes, wheat germ, quinoa, peanuts, carrots, apples, broccoli
Riboflavin (B2)	1.1 mg/day	Oats, quinoa, apple, spinach, tomatoes, rice, salmon
Pyridoxine (B6)	1.3 mg/day	Chickpeas, potatoes, salmon, tuna, cottage cheese, onions, rice, nuts, watermelon
Cobalamin (B12)	2.4 µg/day	Milk, beef liver, beans, strawberry, banana, spinach, salmon, tuna, yogurt, cheese
Methionine	-	Eggs, yogurt, cheese, red meat, soja, milk
Omega 3 fatty acids	1.1 g/day	Chia seeds, sardines, whole bread, milk, beans, salmon, soybean, canola oils, flaxseed
Omega 6 fatty acids	-	Olive oil, safflower, sunflowers oils, peanut oil

Information obtained from the National Institutes of Health (NIH). DRI: Dietary Reference Intake; DFE: dietary folate equivalent.

## Data Availability

Not applicable.

## Abbreviations

SLE	systemic lupus erythematosus
miRNAs	micro RNAs
MTHFR	methylenetetrahydrofolate reductase
DNMTs	DNA methyltransferases
HMTs	histone methyltransferases
HDMTs	histone demethylases
5-methylTHF	5-methyltetrahydrofolate
SAM	S -adenosyl methionine
CDKN3	cyclin-dependent kinase 3
VEGF-C	vascular endothelial growth factor C
IGF-2	insulin-like growth factor-2
PUFAs	polyunsaturated fatty acids
DHA	docosahexaenoic acid
EPA	eicosapentaenoic acid
LA	linoleic acid
